# The Next Frontier in Parkinson's Disease Treatment: A Narrative Review of Innovative Neurosurgical and Gene Therapy Approaches

**DOI:** 10.1002/hsr2.71197

**Published:** 2025-08-27

**Authors:** Muhammad Shaheer Bin Faheem, Muhammad Haroon‐Ul‐Rasheed, Rohma Aftab, Qasra Faheem, Faheem Feroze, Hafiza Qurat Ul Ain, Sumaya Samadi

**Affiliations:** ^1^ Department of Internal Medicine Karachi Institute of Medical Sciences, KIMS Karachi Pakistan; ^2^ CMH Multan Institute of Medical Sciences Multan Pakistan; ^3^ Combined Military Hospital Rawalpindi, CMH Rawalpindi Pakistan; ^4^ Kabul University of Medical Sciences “Abu Ali Ibn Sina” Kabul Afghanistan

**Keywords:** deep brain stimulation, focused ultrasound stimulation, pallidotomy, Parkinson's disease, thalamotomy

## Abstract

**Background and Aims:**

Parkinson's disease (PD) is a progressive neurological condition that consists of both motor and non‐motor symptoms that considerably deteriorate the quality of life. Pharmacological treatments, primarily levodopa, have been used to manage PD. However, side effects such as dyskinesias and motor fluctuations frequently limit their long‐term effectiveness. In recent decades, breakthroughs and innovations in neurosurgical treatments have transformed the entire treatment landscape.

**Methods:**

This narrative review looks at clinical research focused on neurosurgical, gene therapy, and other modern methods for treating PD. Forty‐seven studies were included in the search from PubMed, while three others were taken from other databases.

**Results:**

Deep brain stimulation (DBS) is the most established neurosurgical technique, and has strong evidence that it can improve motor symptoms. Focused ultrasound (FUS) provides a noninvasive option, but the majority of studies related to it still lack long‐term data. Gene therapy strategies like AAV2‐hAADC and ProSavin have shown early‐phase safety and efficacy, but are still in early clinical stages. Newer imaging techniques and robotic surgery can play a critical role PD‐related surgery, in the future.

**Conclusion:**

The range of PD treatment options is growing because of the newer neurosurgical and gene therapy approaches. The current results are promising, but larger‐scale, controlled trials are required to show long‐term safety and efficacy.

## 1 Introduction

Parkinson's disease (PD) is a neurodegenerative condition that is typically expressed in later life as bradykinesia, or an overall slowing of movements, along with additional symptoms of rigidity or resting tremor. Additional symptoms include constipation, sleep disturbances, loss of smell, excessive salivation, mood disorders, and excessive periodic limb movements during sleep [1]. According to the 2015 Global Burden of Disease estimates, PD is the second most common neurodegenerative disease and the one with the fastest growth rate in terms of associated prevalence, disability, and mortality worldwide [2].

Medication therapies like levodopa have been the principal treatment for reducing the symptoms caused by PD, but they were unable to sustain the intended long‐term effects. In PD, levodopa causes refractory tremors, dyskinesias, and fluctuations [3]. During the 1930s, neurosurgeons first used ablative procedures to target the basal ganglia for movement disorders, with evidence linking pathology in the basal ganglia to movement abnormalities and an increased understanding of basal ganglia and subcortical anatomy. Later, in the 1950s and 1960s, lesioning of the globus pallidus (pallidotomy) and the thalamus (thalamotomy) was commonly carried out to treat PD, dystonia and other types of movement disorders [4]. In the early 1990s, deep brain stimulation (DBS), which received FDA permission to treat PD, considerably replaced the role of pallidotomy and thalamotomy [3]. In addition, DBS exhibits symptomatic superiority in comparison to the medical‐only treatment and can help change aberrant neural circuits once it is implanted [5].

This narrative review aims to investigate the latest advancements in neurosurgical therapies for PD and assess how they affect clinical efficacy, safety, and patient outcomes. It discusses advancements in deep brain stimulation technologies, neuromodulation methods, and newer surgical techniques, which can pave the path for future studies and investigations regarding treating PD.

## 2 Methods

The purpose of this narrative review was to examine neurosurgical therapies for Parkinson's disease. PubMed, Scopus, and other databases were used to search the literature for English‐language publications only. “Parkinson's disease,” “deep brain stimulation,” “focused ultrasound,” “pallidotomy,” and associated neurosurgical terms were among the search terms used. Forty‐four of the references were PubMed‐indexed, while the remaining three were taken from Google Scholar or other older archives. The main conclusions were given in a descriptive and comparative style after being organized according to their respective topics. No statistical analysis was applied, as this is a narrative review.

## 3 Evolution of Neurosurgical Techniques

### 3.1 Initial Surgical Techniques

Early in the 20th century, surgery for movement disorders majorly dealt with hyperkinetic disorders in totality rather than treating PD alone. To treat Parkinsonian tremors, Bucy and Case removed the cerebral cortex during the 1930s. However, this kind of ablative surgery caused hemiparesis, and hence, it was eventually abandoned [6]. During surgery on a Parkinsonian patient in 1953, Cooper cut the anterior choroidal artery by mistake and had to ligate it to prevent bleeding. Although the mortality rate was about 10%, the procedure's unexpected and surprising alleviation of tremor and rigidity on the contralateral side led to its increased utilization in patients with PD [7].

### 3.2 Transition From Open to Minimally Invasive Surgeries

During the same period, the works of Spiegel et al. [8] on the advantages of pallidotomy started to appear with the introduction of newer stereotactic surgery. Finally, it was discovered that thalamotomy could reduce Parkinsonian tremor. In conjunction with early stereotaxic procedures, electrical coagulation procedures involving the globus pallidus, thalamus, and ansa lenticularis were also carried out to treat Parkinson's [8]. The absence of medical neurologists' engagement hindered early surgical treatments, raising worries about inaccurate reporting, a lack of long‐term follow‐up, and the possibility of morbidity minimization. A brief halt in the surgical treatment of PD was seen in the 1960s after levodopa was discovered to reduce the symptoms of the condition significantly; this ultimately led to the decline of the surgical procedures of PD [9].

### 3.3 Development and Adoption of Deep Brain Stimulation (DBS)

Finally, a completely different approach for treating the tremors involved stimulating the deep structures in the human brain. Benabid and colleagues' studies, which activated the ventral intermediate nucleus of the thalamus, marked the beginning of the contemporary age of chronic deep brain stimulation (DBS), even though stimulation of the deep parts of the brain had been done before. This stimulation significantly reduced tremors in patients with PD [10].

## 4 Current Techniques

### 4.1 Deep Brain Stimulation

#### 4.1.1 Procedure

Deep brain stimulation is a method that entails the surgical insertion of stimulation leads into specific motor regions of the cortico‐basal ganglia‐thalamo‐cortical circuit, including the subthalamic nucleus (STN), globus pallidus internus (GPi), and ventral intermediate nucleus (ViM) [11]. In DBS, leads and electrodes are coupled to internal pulse generators (IPGs), typically implanted in the subclavicular region, to modify the signals from the leads. The pathological activity is inhibited by high‐frequency electrical oscillations of deep brain stimulation (DBS) aimed at the Subthalamic nuclei or GPi areas, thereby alleviating or diminishing the devastating clinical manifestations of PD, including tremors, stiffness, and bradykinesia [12].

#### 4.1.2 Indications for DBS

There are three major indications for the application of DBS in the treatment of PD. The primary indication is for patients with medication‐resistant tremors. The second significant indication for DBS encompasses patients experiencing difficulties with long‐term levodopa medication, such as levodopa‐induced dyskinesias and the wearing‐off phenomenon associated with it. Additionally, it serves as an impressive alternative for patients who may exhibit intolerance to dopaminergic medications such as levodopa [13].

#### 4.1.3 Outcomes

The subthalamic nucleus and globus pallidus internus in our brain are the predominant targets for deep brain stimulation. The Unified Parkinson's Disease Rating Scale (UPDRS) is considered the benchmark for evaluating the severity and development of PD. Numerous researchers utilize the UPDRS to evaluate and differentiate the effects of STN versus GPi DBS. These studies indicate that STN DBS and GPi DBS yield similar results and are effective in tremor reduction, gait improvement, and improving undesirable effects, including mood alterations and apathy [14]. DBS has some known risks; a meta‐analysis of 11 studies including 1368 patients found a 21% incidence of postoperative delirium (POD) in PD patients undergoing DBS. Older age, lower cognitive function, and higher non‐motor symptom burden were the main predictors of POD. In contrast, gender, motor symptoms, and comorbidities showed no clear association. These findings suggest that cognitive and non‐motor assessments may help us identify those at risk of complications before DBS surgery [15].

### 4.2 Lesioning Procedures

#### 4.2.1 Pallidotomy

##### 4.2.1.1 Indications

• Parkinson's disease: Complications arising from disease progression and medical treatment, encompassing tremors, wearing off, motor fluctuations, and dyskinesia in patients who exhibit a favorable response to levodopa medication. Pallidotomy should ideally be unilateral in patients with PD.

• Dystonia: Debilitating symptoms unresponsive to pharmacological treatment, including anticholinergics, benzodiazepines, and botulinum toxin. Pallidotomy may be conducted bilaterally in patients with dystonia in specific instances [16].

##### 4.2.1.2 Procedure

Pallidotomy is an invasive technique that can be performed unilaterally or bilaterally and requires making a lesion by a minimally invasive surgery in the globus pallidus inside our brain [17].

##### 4.2.1.3 Outcomes

Pallidotomy is curative for all primary motor symptoms of PD, including tremor, rigidity, and bradykinesia, as well as movement disorders and drug‐induced dyskinesias and dystonia. It may also improve axial symptoms, including coordination, balance, and freezing episodes [18].

The improvement of axial symptoms post‐unilateral pallidotomy is less reliable than that of appendicular symptoms, with numerous patients experiencing a decline after some time. To achieve consistent benefits for axial symptoms, bilateral procedures are necessary; however, bilateral pallidotomy is linked to a high incidence of hypophonia. Several cases have reported urinary incontinence and cognitive deterioration related to bilateral pallidotomy [18].

Two prevalent reasons for patients' lack of recovery after pallidotomy are: (1) the patient has Parkinsonian syndrome rather than idiopathic PD, or (2) the lesions are located outside the sensorimotor territory of the globus pallidus internus (GPi) [18].

### 4.3 Thalamotomy

#### 4.3.1 Indications

• Essential tremor: A debilitating, primarily unilateral kinetic tremor of the upper extremities, persisting despite adequate pharmacological intervention, including beta‐blockers, primidone, and benzodiazepines [16].

• Parkinson's disease: Unilateral resting tremor unresponsive to pharmacological intervention in tremor‐predominant cases [16].

• Cerebellar outflow tremor: Medically resistant unilateral kinetic, postural, or resting tremor resulting from multiple sclerosis or trauma to the brain [16].

#### 4.3.2 Procedure

The surgical removal of a portion of the thalamus is known as thalamotomy. Usually, it is done to treat movement disorders like essential tremors and PD. A permanent and irreversible lesion is produced by thalamotomy. The ventral intermediate nucleus across from the injured limb is typically the target. In the end, this aids in managing symptoms associated with PD [19].

#### 4.3.3 Outcomes

Thalamotomy is highly effective for the management of Parkinsonian tremor. Lesions are typically situated in the cerebellar receiving region, ventralis intermedius (Vim). If the lesion extends anteriorly into the basal ganglia receiving area, thalamotomy could improve rigidity and reduce drug‐induced dyskinesias, yielding better results.

However, it is ineffective for bradykinesia, freezing, postural instability, or the gait disorder associated with PD [18].

Bilateral thalamotomy is linked to an excessively high rate of speech complications. This typically presents as aphasia, dysarthria, or dysphonia. Bilateral thalamotomy is not advised, given the presently available deep brain stimulation (DBS) techniques, unless alternative methods are contraindicated. Due to the ineffectiveness of bradykinesia and the possibility of exacerbating gait issues with thalamic surgery, stimulation of the subthalamic nucleus (STN) or globus pallidus (GPi) is more frequently advised [18].

#### 4.3.4 Sub Thalamotomy

The Sub‐thalamic nucleus is a crucial part of basal ganglia, as it is linked to numerous other structures within the basal ganglia and the different nuclei. Overactivity of these primary symptoms of PD and dyskinesias. Subthalamotomy serves as an alternative surgical option for individuals whose conditions are resistant to pharmacological treatment or who cannot undergo DBS implantation due to medical contraindications or accessibility issues. Numerous studies have evidenced that subthalamotomy daily levodopa requirements are linked to minimal, primarily transient, complications. However, still little clinical evidence to justify its application as a treatment modality for PD [20].

### 4.4 Focused Ultrasound Stimulation (FUS)

#### 4.4.1 Focused Ultrasound Stimulation (FUS)

Focused ultrasound stimulation (FUS) is a nonionizing neuromodulatory method that utilizes sound waves to transiently and reversibly alter the electrical activity of deep brain structures. It is presently under investigation as a prospective breakthrough treatment for PD [21].

#### 4.4.2 Magnetic Resonance‐Guided Focused Ultrasound (MRgFUS)

Focused ultrasound, an ablative, incision‐less approach that was first shown to be safe in animal models such as rabbits and other primate species, is a newer method for treating PD [22]. MRgFUS, an entirely transcranial imaging‐guided procedure, is performed nowadays and involves a helmet equipped with roughly 1000 separate piezoelectric transducers. By spreading the energy across the large cranial surface and using degassed cold water to cool the scalp between the helmet and skull surface, these transducers deliver an epicenter of concentrated ultrasonic mechanical energy to a small brain tissue target that is absorbed and converted into heat. This prevents significant heating. Software that adjusts for variations in cranial bone thickness or elements that can cause wave distortion, like acoustic impedance, is linked to the array of transducers [23]. Consequently, this technique makes it possible to precisely deliver transcranial ultrasonic mechanical energy to the desired nucleus, creating tiny lesions. Low frequency and, thus, low‐temperature ultrasonic energy discharges are applied in confined tissular zones that act as controls for pre‐planned targeted sites before the formation of permanent lesions. The temperature is raised until the ablative threshold is reached after the target has been clinically confirmed, producing a permanent lesion [24].

#### 4.4.3 Indications for Magnetic Resonance‐Guided Focused Ultrasound (MRgFUS)

To be considered a candidate for this treatment, a skull density ratio greater than 0.4 must be established, taking into account the variation in skull thickness among patients. Considering the permanent nature and lack of real‐time adjustability of ablative therapies, such as radiofrequency ablation is necessary. Advanced PD patients who are unsuitable candidates or who decline to undergo DBS are eligible for MRgFUS. In addition to the potential for patients with MRI‐incompatible implants, patients with implants from prior neurosurgeries or skull abnormalities might not qualify as suitable candidates for FUS [24].

#### 4.4.4 Clinical Outcomes

MR‐guided focused ultrasound has been used to try subthalamotomy for asymmetrical PD, but there has been a higher rate of side effects, which is concerning. In a prospective, open‐label pilot study, Martínez‐Fernández et al. [25]. included ten patients with PD who were significantly asymmetric, had a poor response to medication, refused deep brain stimulation (DBS), had a situation in which bilateral DBS was not recommended, or were borderline eligible for DBS. At 6 months, there was a 53% improvement in MDS‐UPDRS in the off‐medication state and a 47% improvement in the on‐medication state. There were 38 reported side effects at the 6‐month follow‐up, but none of them were considered severe. Regardless of the medication state, three of the seven side effects that persisted at the time were linked to subthalamotomy: subjective speech disturbance and dyskinesia in the treated arm. In one patient who was taking medication, these side effects disappeared after the levodopa dosage was decreased, and in the patient who was not taking medication, they almost went away on their own within 6 months. Subthalamotomy‐related transient side effects included elevated blood pressure during the procedure in 50% of cases, pin site head pain in 60% of cases, and gait ataxia in 60% of cases. Ten percent of cases had temporary facial asymmetry, and 20% had moderate impulsivity [26]. However, the absence of a control group and the high incidence of side effects like dyskinesia and speech disturbance, limit the generalizability of the above results. In a double‐center, double‐blind, sham‐controlled pilot randomized trial involving 27 patients with tremor‐dominant PD, hand tremor, as measured by the Clinical Rating Scale for Tremor A + B subscores, improved by 62% from baseline after focused ultrasound thalamotomy. The adverse effects observed during FUS were divided into two categories: thalamotomy‐related and MRI/US environment‐related. The most common side effects associated with thalamotomy were ataxia (35%), oral paresthesia (27%), and finger paresthesia (39%). Paresthesia lasted up to a year in 19% of patients, and ataxia occurred in 4% [24]. Finally, a multicenter open‐label trial evaluated the safety and therapeutic value of unilateral focused ultrasound (FUS) of the globus pallidus internus (GPi) in 20 patients with PD who exhibited responsiveness to levodopa, levodopa‐induced dyskinesia, significantly asymmetrical motor symptoms, and a minimum 30% disparity in motor scores on the Movement Disorders Society version of the United Parkinson's Disease Rating Scale between the on and off medication states. The authors anticipated enhancement through unilateral intervention. Total Unified Dyskinesia, Rating Scale scores exhibited a 59% improvement at 3 months post‐MRgFUS, with this improvement maintained throughout the study duration of 12 months. Motor symptoms, assessed by MDS‐UPDRS III, improved by 44.5% at 3 months and 45.2% at 12 months relative to baseline. Dysarthria (20%), fine motor deficits (10%), visual field deficits (5%), cognitive disturbances (5%), and facial weakness (5%), among other adverse neurological effects, were typically mild and temporary [27]. While the above findings were promising, the study lacked a control group, and several patients experienced temporary neurological effects.

### 4.5 Cost Effectiveness

Mahajan et al. conducted a meta‐analysis reviewing 109 articles encompassing 3573 patients, comparing the cost‐effectiveness of radio‐frequency ablation and deep brain stimulation (DBS) while also predicting the threshold that focused ultrasound (FUS) must surpass to be deemed the most cost‐effective treatment. This meta‐analysis identified DBS as the most cost‐effective intervention over a 22‐month follow‐up, estimating an adverse effects rate of 16.2% based on treatment costs. Utility values derived from Ravikumar et al [28]. indicated that percent reduction thresholds in UPDRS‐III‐Off scores of 15.5% and 32.8% are necessary to attain cost‐effectiveness over two and 5‐year periods, respectively [22].

### 4.6 Gene Therapy

Although gene therapy is not a classical neurosurgical technique, it often involves stereotactic delivery and falls under the broader umbrella of neuromodulation for PD. Complementary DNA (cDNA) sequences encoding genes implicated in the pathophysiology of PD are delivered via a viral vector as part of gene supplementation therapy. Gene therapies seek to either improve the health of dopaminergic neurons by maintaining and restoring neurotrophic factors or improve the bioavailability of dopamine (DA) in the nigrostriatal pathway by directly enhancing the enzymes responsible for dopamine production [29]. Table 1 below highlights the major gene therapy‐related trials.

**Table 1:** Summary of key gene therapy‐related trials for Parkinson's disease.

Study/trial	Therapy	Sample size	Study design	Key findings
AAV2‐GAD Phase II	Gene therapy (STN)	45	Double‐blind RCT	Improved motor UPDRS, reduced dyskinesia
ProSavin	Gene therapy (putamen)	15	Open‐label, Phase I/II	Decrease in UPDRS scores over 2 years
AAV2‐hAADC	Gene therapy	6–10	Open‐label pilot	Increased dopamine turnover, reduced UPDRS

## 5 Types of Gene Therapy, Clinical Trials, and Outcomes

### 5.1 AAV2‐GAD Gene Therapy

Inhibition is lost when GABA input to the STN is reduced due to the death of dopaminergic neurons present in the substantia nigra. It has been discovered that muscimol (GABA) agonist infusion into the STN can momentarily alleviate Parkinsonian symptoms. The key enzyme for GABA synthesis is glutamic acid decarboxylase (GAD), and gene therapy—which involves inserting the GAD gene into an adeno‐associated viral vector (AAV2)—has been successfully applied in human and animal research. Delivering GAD to the STN is thought to normalize output from the STN and restore GABA transmission within the STN. In a phase I open‐label trial involving 12 patients, unilateral infusion of AAV2‐GAD into the STN was found to be safe and showed some motor improvements; however, the small sample size and lack of a control group limit the strength of these results [30]. At 6 and 12 months, a phase II trial with 45 patients tested the treatment in a randomized, double‐blind study. The results showed improvements in motor symptoms and dyskinesia. However, it is to be noted that the responses had variations among the participants [31]. In addition, the study only followed patients for a short time period; therefore, larger trials are needed to confirm these results.

### 5.2 LV‐GCH1‐TH‐AADC

A new lentiviral vector called ProSavin expresses TH, AADC, and GCH1. A long‐term safety and efficacy study assessing the same patient group is currently underway, while the phase ½ open‐label, dose‐escalation study has concluded [32, 33]. In an open‐label, dose‐escalation phase I/II study, which included 15 patients, ProSavin reduced UPDRS‐III scores at 2‐year follow‐up. However, the absence of a placebo and the small size of the study show limitations in the results [32, 33]. Ninety‐one per cent of drug‐related adverse events in subjects administered ProSavin were minimal and/or manifested during a 1‐year posttreatment period. Dyskinesia, acute psychosis, and indeterminate nervous system ailments were among the three therapy‐related serious complications [32].

### 5.3 AAV2‐hAADC

Numerous gene therapy research has concentrated on enhancing the expression of dopamine synthesis enzymes, specifically tyrosine hydroxylase (TH), l‐amino acid decarboxylase (AADC), and GTP cyclohydrolase (GCH1) [34].

In one study, advanced PD individuals were evaluated for the safety and effectiveness of intraputaminal infusion of the adeno‐associated serotype two viral vectors encoding human AADC (AAV‐hAADC‐2). Patients in these small open‐label trials showed improvement in UPDRS scores at 6 months [34]. It is to be noted that the conclusions are limited by sample size and short follow‐up durations. In a different study, participants with advanced PD were given intraputaminal injections of adeno‐associated viral vector (AAV‐hAADC) at low or high doses. Patients' mean improvements in the off‐state and on‐state UPDRS‐III scores at 6 months were 36% and 28%, respectively [35].

According to the two aforementioned studies, AADC gene therapy for PD has better safety and tolerability [34, 35].

### 5.4 Neurotrophic Signaling Restoration

Patients with PD exhibit a deficiency of intrinsic neuronal growth factors alongside the degeneration of dopaminergic neurons [29]. At present, gene therapy utilizing AAV2‐GDNF is being actively studied in two clinical trials. It concluded that the phase 1 study results increased by 36% and 54% in 18F‐DOPA putaminal signal at 6‐month and 18‐month follow‐ups, respectively [29]. Although imaging suggested increased putaminal 18F‐DOPA uptake in a phase I study, there was no significant clinical improvement in UPDRS scores, highlighting a lack of connection between the biomarker and the clinical efficacy [29].

### 5.5 Patient Considerations for Gene Therapy

Most Parkinson's medications work by boosting dopamine levels through three approaches: increasing its production, prolonging its activity, or slowing its breakdown [36]. As genetic testing becomes increasingly accessible, it is plausible to contemplate the selection of patients for specific therapies which are based on their genotype. Individuals exhibiting the advanced Parkinsonian phenotype with movement and motor fluctuations are anticipated to derive the most significant advantage from a gene therapy strategy designed to enhance dopamine synthesis. It's also essential to take into account that gene therapy is unchangeable, especially for patients with severely advanced PD, who pose more vulnerability to side effects like ICD and dopamine dysregulation syndrome [37, 38].

**Figure 1:**
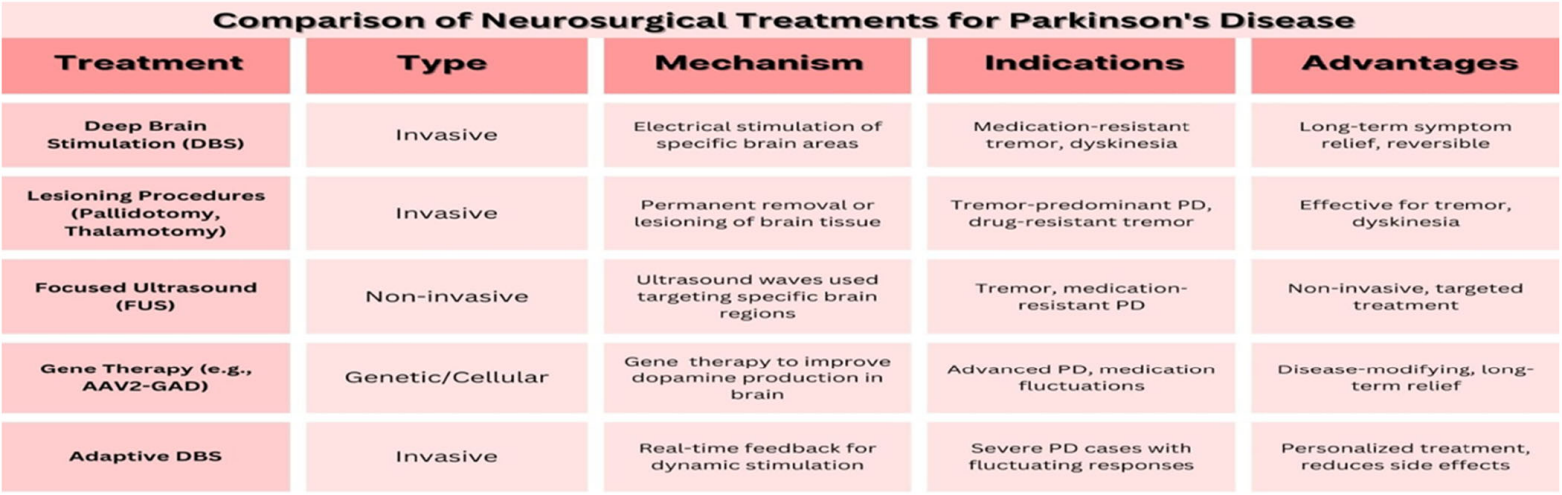
Comparison of different therapies for Parkinson's disease.

Different types of therapies for PD have been discussed in Figure 1.

## 6 Innovations in Neurosurgery

### 6.1 Advanced Imaging Techniques

Advanced neuroimaging is essential for the early detection and management of neurodegenerative diseases such as PD. Advancements in MRI, PET, and other imaging techniques have enhanced our capacity to identify minor alterations in the brain, facilitating early therapies. The integration of AI demonstrates the potential to improve early detection methods. Resolving these difficulties is crucial for optimizing the potential of neuroimaging developments [39].

### 6.2 Proton Magnetic Resonance Spectroscopy in Parkinson's

PD is a neurodegenerative disorder that poses challenges for early diagnosis, underscoring the need to decelerate disease progression and enhance treatment efficacy. In vivo, molecular resonance imaging (MRI) has been recognized as an effective imaging biomarker for early differential diagnosis and assessment of treatment efficiency. Proton Magnetic Resonance Spectroscopy (MRS) exhibits strong test‐retest reliability, is noninvasive and cost‐effective, and does not necessitate contrast chemicals for molecular imaging. It is not confined to specialized centers and can be expanded to regular public health facilities. Recent advancements in magnetic resonance spectroscopy, including enhanced magnetic fields and dependable techniques for absolute metabolite quantification, have facilitated in vivo insights into PD pathology. The decreased NAA levels within cortical‐basal ganglia networks indicate neuronal death and mitochondrial metabolic impairment in PD. Alterations in Glutamate and GABA levels observed in PD patients may indicate impairment of movement‐related neuronal excitatory and inhibitory functions. Proton Magnetic Resonance Spectroscopy has demonstrated great benefit in the differential diagnosis and treatment related to PD [40].

### 6.3 Role of High‐Resolution MRI and Diffusion Tensor Imaging (DTI) in Surgical Planning

Brain MRI is essential for identifying cerebrovascular injuries and measuring brain atrophy in individuals with Parkinsonism. It aids in identifying structural lesions that contribute to Parkinsonism, gait disturbances, and tremors and should be included in the differential diagnosis of PD versus other forms of Parkinsonism. MRI can detect structural abnormalities linked to alternative causes of Parkinsonism, such as vascular pathology or neoplasms. It can also quantify the extent and location of cerebral atrophy. Abnormal T2 MRI hypointensities in the putamen can differentiate MSA‐P from PD with 88% sensitivity and 89% specificity [41]. Diffusion tensor imaging (DTI) is an MRI modality that quantifies water diffusion in tissue to provide neural tract pictures [42]. initially proposed it in 1991, and in 1992, it was utilized to generate the inaugural DTI image depicting neuronal tracts traversing the brain. DTI has attracted attention because of its capacity to provide information about brain fiber and lesions. This information is essential for preoperative neurosurgical planning [43].

### 6.4 Concept of Real‐Time Feedback and Adaptive Stimulation

Brain‐computer interfaces (BCIs) can be used to analyze pathological brain signals in individuals who have advanced PD and control therapeutic deep brain stimulation (DBS). A research study evaluated BCI‐controlled adaptive deep brain stimulation (aDBS) in eight PD patients, utilizing input from local field potentials captured by stimulation electrodes. Compared to traditional continuous stimulation (cDBS), motor scores improved by 29% and 27% during aDBS, respectively, by 66% (unblinded) and 50% (blinded). This resulted in a 56% decrease in stimulation time and a concomitant reduction in energy consumption. Adaptive DBS demonstrated superior efficacy compared to both no stimulation and random intermittent stimulation. The research indicated that BCI‐controlled DBS was more accessible and could surpass traditional continuous neuromodulation in efficiency and effectiveness for PD [44]. These outcomes need to be confirmed by future larger trials.

### 6.5 Robotic‐Assisted Surgery

Deep brain stimulation (DBS) is a surgical technique that inserts stimulation leads into specified motor areas of the cortico‐basal ganglia thalamo‐cortical circuit, including the subthalamic nucleus, globus pallidus internus, and ventral intermediate nucleus. This technique, combined with internal pulse generators, suppresses abnormal brain activity, potentially reducing the clinical manifestations of PD [11, 12]. Furthermore, Deep Brain Stimulation (DBS) robots, including Neuromate, Surgiscope, ROSA, and Renaissance, are employed in the operating room. Finally, the introduction of surgical robots has already made a significant contribution [45].

### 6.6 Application in Electrode Placement and Precision

#### 6.6.1 Methods

The research included patients who received a preoperative MRI while under general anesthesia. The trajectory planning and MRI were conducted the day before surgery for the other patients. The individual was positioned using a head clamp tailored for intraoperative CT, and measurements were obtained for the Renaissance robot attachment base placement. The intraoperative CT was acquired in a sterile manner and integrated with the preoperative MRI. The robot's base position was established in relation to the target trajectories, referred to as the fiducial CT. In addition, The MRI‐CT fusion procedure was finalized in the software, and the robot was affixed to the base for the burring and positioning of the target cannula. A secondary intraoperative CT was conducted with the robot affixed and the cannula positioned, and the discrepancy between the placement of the cannula and the preoperative trajectory plan was evaluated [46].

#### 6.6.2 Precision

A study compared the accuracy of a robotic stereotactic platform for deep brain stimulation (DBS) lead implantation with that of conventional frame‐based systems. The study examined 126 sequential DBS lead placement methods utilizing a robotic stereotactic platform, including PD. The findings indicated a mean radial error of 1.06 mm in intraoperative fluoroscopic computed tomography, with a mean operative duration of 238 min for an asleep, bilateral implant without the placement of an implantable pulse generator and 116 min for skin‐to‐skin procedures. The study presented a simplified sequence for DBS lead placement using robot‐assisted stereotaxy, achieving almost similar accuracy, eliminating the requirement for coordinate checking, reducing human error, and facilitating training [47].

### 6.7 Benefits of Robotic‐Assisted Surgery

A meta‐analysis was conducted, which assessed the efficacy and safety of robot‐assisted deep brain stimulation (DBS) surgery for individuals with PD. The analysis encompassed 15 research studies covering 732 people diagnosed with PD. The results indicated a notable decline in vector error, complication rate, and a reduction in levodopa‐equivalent daily dose (LEDD). In addition, there were improvements in UPDRS, UPDRS III, and UPDRS IV scores. The study concluded that robot‐assisted DBS is a safe and reliable treatment alternative, providing superior precision relative to traditional frame‐based stereotactic methods [48].

### 6.8 Optogenetics and Other Emerging Techniques

Comprehending the mechanisms that govern the efficacy of DBS is essential for enhancing current methodologies across diverse patient populations and for expanding future applications. However, such advancements cannot solely arise from clinical trials, as various factors constrain opportunities for experimentation. Improvements in DBS are being propelled by findings from research utilizing animal models, where neural circuits can be mapped and controlled with exceptional specificity through a technique known as optogenetics. Optogenetics is an emerging technique that enables the control of neural activity through opsins, which are light‐sensitive ion channels. The expression of these channels can be targeted to specific neuronal populations of interest. These genes are frequently delivered using adeno‐associated viruses (AAV), from which pathogenic genes have been excised and substituted with genes that encode excitatory opsins (e.g., channelrhodopsin, ChR2) or inhibitory opsins (e.g., halorhodopsin, NpHr) [49].

#### 6.8.1 Potential Future Applications in Parkinson's Disease

**Figure 2:**
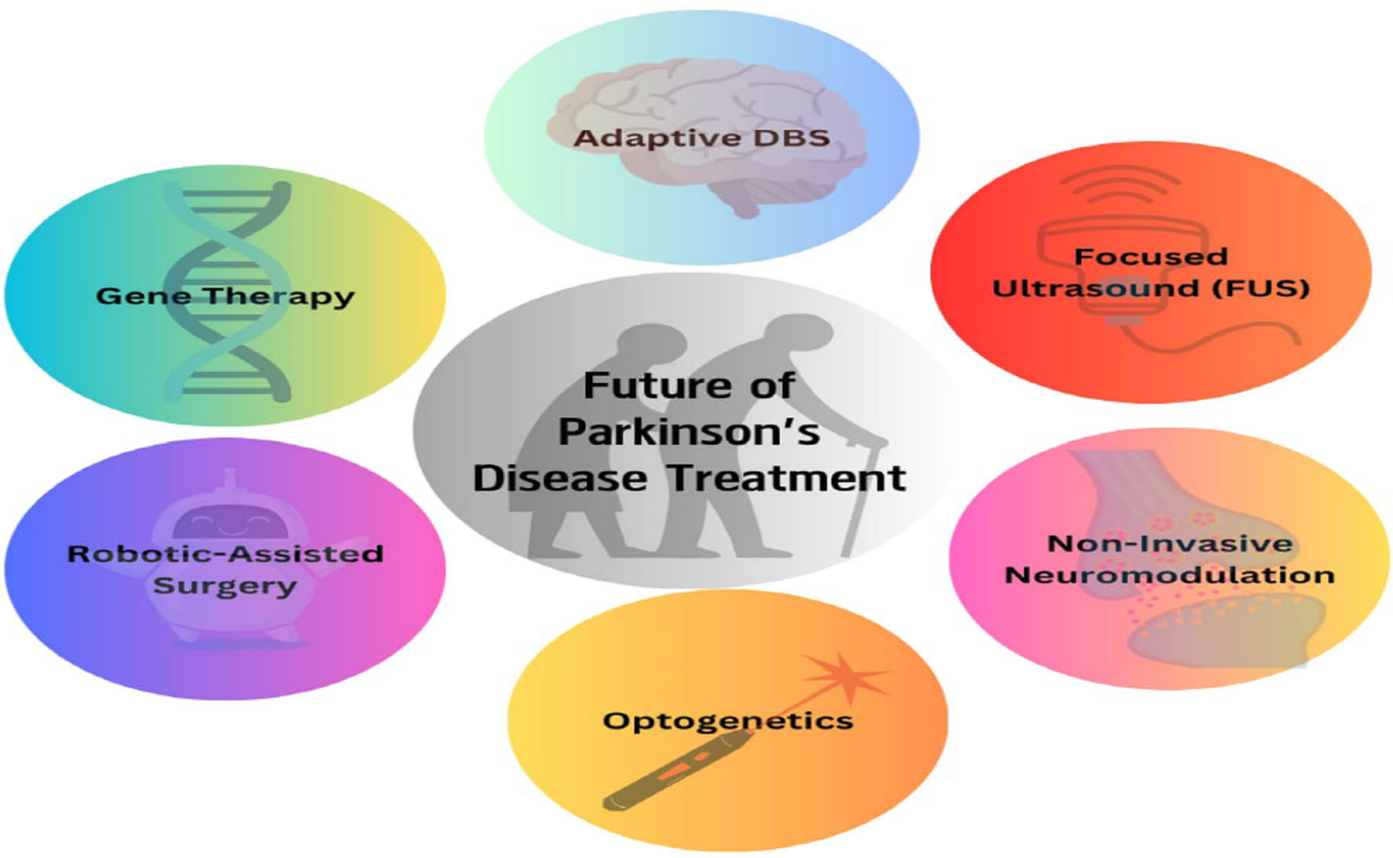
Emerging treatment options for Parkinson's disease.

New developments in technology and neuroscience are changing how PD is treated in the future. Current treatments frequently focus on symptom control, leaving a significant gap for strategies that address the underlying pathology and improve long‐term outcomes. In the following paragraphs, we discuss some possible future transformative applications. Figure 2 further depicts the potential future applications in PD.

#### 6.8.2 Adaptive and Closed‐Loop Neuromodulation

Adaptive DBS adjusts stimulation in response to real‐time brain signals, showing improved motor outcomes and the overall energy efficiency [44]. The use of AI could improve stimulation parameters even further, solidifying this strategy as a pillar of precision medicine in the treatment of PD.

#### 6.8.3 Innovations in Imaging and Surgical Precision

Advanced imaging techniques like DTI and high‐resolution MRI can potentially refine targeting for future neurosurgical procedures [43]. These technologies can potentially guide lesioning procedures such as MR‐guided Focused Ultrasound (MRgFUS) and increase the accuracy of electrode placement in DBS applications. To ensure ideal targeting of the lesion and reduce complications, future advancements might include real‐time imaging during surgeries. Additionally, by lowering human error and improving results, the use of robotic‐assisted platforms can improve precision in neurosurgical procedures.

#### 6.8.4 Noninvasive Neuromodulation Techniques

MRgFUS can assist in future bilateral and robotic‐assisted interventions in PD [24]. Furthermore, the use of robotic‐assisted platforms, such as ROSA [45], can improve precision in neurosurgery procedures, lowering human error and improving results.

#### 6.8.5 Gene Therapy for Disease Modification

Gene therapy can offer long‐term neuroprotection and disease modification, especially when used according to the patient's genotype [30, 31]. These treatments provide hope for long‐term symptom reduction and neuroprotection, potentially halting disease progression. Future developments might concentrate on modifying gene therapy strategies according to phenotypic and genetic characteristics to increase their efficacy.

#### 6.8.6 Emerging Technologies in Neuromodulation

Optogenetics is an emerging experimental technique that modulates brain circuits with remarkable accuracy [49]. Preclinical research to restore motor function in PD animals through optogenetics in human applications is still in the early stages. Although it is in the preclinical stage, in the future, optogenetics can completely change the way neuromodulation is administered.

#### 6.8.7 Multimodal and Combination Therapies

Combining treatments like adaptive DBS, gene therapy, and neuroprotective medications to address different aspects of the illness may be the way of the future for managing PD. By treating neurodegeneration at its root, this multimodal strategy may have synergistic effects that improve both motor and non‐motor symptoms. Together, these advancements can help us shift from symptomatic management to disease modification in PD.

#### 6.8.8 Noninvasive Vagal Nerve Stimulation (nVNS)

Noninvasive vagal nerve stimulation (nVNS) is an emerging neuromodulatory approach for treating Parkinson's disease. By stimulating the vagus nerve, it can influence central autonomic and motor circuits. A systematic review was conducted across 10 studies involving 217 patients. While nVNS showed marginal improvement in freezing of gait (FOG), it demonstrated no major improvement in UPDRS scores. This treatment appeared safe, with no serious adverse effects reported [50]. Although nVNS presents a promising noninvasive approach, large‐scale trials are needed to better understand its potential role in PD management for the future.

## 7 Conclusion

In conclusion, developments in neurosurgical methods, gene therapy, and noninvasive technology are causing a revolutionary change in treating PD. New neurosurgical and gene‐based treatments are starting to change how we treat PD. Deep brain stimulation remains the most dependable approach, with substantial evidence to back its usage. Focused ultrasound offers a promising, less‐invasive option, although current research is limited and short‐term in nature. Gene therapy can show long‐term solution, but it is still in its early stages and requires additional research. The combination of these newer techniques with modern imaging and robot‐assisted surgery illustrates how PD can be tackled in a completely new way. As these alternate approaches evolve, selecting the proper patients and making these treatments more accessible will be equally crucial as developing the science. Future trials and research will pave the path for a personalized approach for the treatment of PD.

## Author Contributions

**Muhammad Shaheer Bin Faheem:** conceptualization, writing – original draft, writing – review and editing, data curation, supervision, resources, project administration, validation, and visualization. **Muhammad Haroon‐Ul‐Rasheed:** visualization, writing – original draft, investigation, methodology, and validation. **Rohma Aftab:** investigation, writing – original draft, and visualization. **Qasra Faheem:** resources, validation, writing – original draft, and methodology. **Faheem Feroze:** validation, writing – original draft, and resources. **Hafiza Qurat Ul Ain:** investigation. **Sumaya Samadi:** investigation.

## Ethics Statement

The authors have nothing to report.

## Consent

The authors have nothing to report.

## Conflicts of Interest

The authors declare no conflicts of interest.

## Transparency Statement

The lead author, Muhammad Shaheer Bin Faheem, affirms that this manuscript is an honest, accurate, and transparent account of the study being reported; that no important aspects of the study have been omitted; and that any discrepancies from the study as planned (and, if relevant, registered) have been explained.

## Data Availability

Data sharing not applicable to this article as no data sets were generated or analyzed during the current study.
